# Prescribing Clinicians’ Perspectives on Evidence-Based Psychotherapy for Posttraumatic Stress Disorder

**DOI:** 10.3390/bs4040410

**Published:** 2014-10-21

**Authors:** Erin R. Barnett, Nancy C. Bernardy, Aaron B. Jenkyn, Louise E. Parker, Brian C. Lund, Bruce Alexander, Matthew J. Friedman

**Affiliations:** 1 Department of Psychiatry, Geisel School of Medicine at Dartmouth, 1 Rope Ferry Road, Hanover, NH 03755, USA; E-Mails: Nancy.Bernardy@va.gov (N.C.B.); Matthew.Friedman@dartmouth.edu (M.J.F.); 2 Dartmouth Trauma Interventions Research Center, One Medical Center Dr., Lebanon, NH 03756, USA; 3 National Center for Posttraumatic Stress Disorder, Veterans Affairs Medical Center, 215 North Main Street, White River Junction, VT 05009, USA; E-Mail: Aaron.Jenkyn@va.gov; 4 Department of Management and Marketing, College of Management, University of Massachusetts, Boston, 100 Morrissey Boulevard, Boston, MA 02125, USA; E-Mail: Louise.Parker@umb.edu; 5 Center for Comprehensive Access & Delivery Research and Evaluation, Iowa City VA Health Care System, Mailstop 152, 601 Hwy 6 West, Iowa City, IA 52246, USA; E-Mails: Brian.Lund@va.gov (B.C.L.); Bruce.Alexander@va.gov (B.A.); 6 Department of Pharmacology & Toxicology, Geisel School of Medicine at Dartmouth, 1 Rope Ferry Road, Hanover, NH 03755, USA

**Keywords:** evidence-based practice, posttraumatic stress disorder (PTSD), pharmacology, implementation barriers, clinical practice guidelines, Veteran’s Affairs

## Abstract

Evidence-based psychotherapies (EBP) for Posttraumatic Stress Disorder are not utilized to their full extent within the Department of Veterans Affairs (VA). VA provides care to many persons with PTSD and has been in the forefront of clinical practice guidelines and EBP training and dissemination. Yet VA continues to find EBP implementation difficult. Veterans with PTSD often initially present to prescribing clinicians, who then help make care decisions. It is therefore critical that these clinicians correctly screen and triage appropriate mental health care. The purpose of this study was to assess VA prescribing clinicians’ knowledge, perceptions, and referral behaviors related to EBPs for PTSD and to identify facilitators and barriers to implementing EBPs within VA. We conducted qualitative interviews with 26 VA prescribing clinicians. Limited access to EBPs was the most commonly noted barrier. The clinicians we interviewed also held specific beliefs and behaviors that may delay or deter EBPs. Strategies to improve utilization also emerged. Findings suggest the need for increased access to EBPs, training to optimize the role of prescribing clinicians in helping Veterans with PTSD make appropriate care decisions, and specific organizational changes to facilitate access and effective referral systems for EBPs.

## 1. Introduction

Posttraumatic stress disorder (PTSD) is associated with high rates of comorbid physical and mental health conditions [1,2] and considerable functional impairment [3]. Because PTSD is the most common mental health diagnosis among US Veterans accessing the Department of Veterans Affairs (VA) services [4], the VA has served as a leader in promoting appropriate evidenced based treatments. These efforts have included: (a) publication of the VA/Department of Defense (DoD) Clinical Practice Guideline for PTSD in 2010 [5], which recommends first-line psychosocial and pharmacological interventions for PTSD; (b) systematic PTSD screening; (c) symptom monitoring; (d) timely delivery of mental health visits; and (e) the requirement that PTSD clinics offer one of two evidence-based psychotherapies (EBPs), Cognitive Processing Therapy or Prolonged Exposure [6], to Veterans diagnosed with PTSD.

To date, over 6100 VA therapists have received training in either Cognitive Processing Therapy or Prolonged Exposure [7]. These individual EBPs are cognitive-behavioral therapies, time-limited, and highly effective: Remission rates of the EBPs are approximately 60% with improvements observed in both civilians and Veterans [8,9]. EBPs are not accompanied by the side effects of medications. Further, although long-term outcomes have not yet been studied in Veterans with PTSD, treatment gains have sustained over time in other adult populations with PTSD [10]. People with PTSD may also prefer psychosocial treatments over medication. For example, in separate studies, trauma-exposed women and Veterans with PTSD rated descriptions of Prolonged Exposure as more desirable than first-line medications (e.g., antidepressants) for PTSD [11,12].

Despite VA’s efforts and the efficacy of individual EBPs, most Veterans with PTSD either do not receive any EBPs at all or do not receive them for sufficient durations; an estimated 6.3% to 33% of Veterans with PTSD within VA receive a minimally adequate length of an EBP [13,14,15]. The challenge of promoting evidence-based care is not limited to PTSD or to the VA [16]. The health services literature describes many organizational challenges preventing access to evidence-based healthcare generally, including lack of resources and systems to sustain their use [17]. Researchers have identified challenges specific to EBPs for PTSD, including patient barriers to seeking help, therapist resistance to EBPs, and organizational barriers to implementation [18,19,20].

Prescribing clinicians are central to patients’ ability to access EBPs for PTSD within the VA. Many Veterans with PTSD initially present to prescribing clinicians, who then manage referrals to other services such as EBPs. In addition, patients and other providers hold prescribing clinicians and their care recommendations in high esteem. For these reasons, it is critical to understand clinical prescribers’ perspectives of EBPs to improve the implementation of these highly beneficial treatments. We interviewed primary care physicians, psychiatrists, Advanced Practice Registered Nurses, and Doctors of Pharmacy, all prescribing clinicians in VA in an effort to understand their knowledge, perceptions, and referral behaviors related to EBPs for PTSD. Specifically, we sought to determine if they were aware of the treatments the VA/DoD PTSD guideline recommends and the availability of these treatments in their facilities. We also examined whether prescribing clinicians effectively conveyed treatment options to patients and made collaborative treatment decisions with them. Finally, we asked prescribing clinicians about what barriers and facilitators they perceived to the adoption of EBPs for PTSD in their facilities.

## 2. Method

### 2.1. Facility Selection

The researchers selected VA Medical Centers (VAMC) based on facility-level data reflecting prescribing practices of PTSD patients from our larger work [21]. Facilities were grouped into quartiles based on absolute change in PTSD psychotropic prescribing patterns during the study period (1999–2009). Previous research showed that rural residents had an increased likelihood of receiving benzodiazepines [21], therefore at least one facility selected within each category was rural, as classified by the Urban, Rural, Highly Rural Classification system. PTSD patient visit volume was also taken into account, with an effort to select facilities with both high and low numbers. Finally, facilities were also selected to represent specific geographic regions. In total 12 VA Medical Centers and 2 Community Based Outpatient Clinics were selected for inclusion in the study. Prescribing clinicians from 1 facility did not respond to recruitment e-mails, leaving 13 facilities in the final sample.

### 2.2. Participant Selection

The researchers asked mental health team leaders to provide the names of prescribing clinicians who treat PTSD in their mental health (MH) and primary care (PC) clinics. One MH and one PC prescribing clinician were then randomly invited from each of the 12 facilities to be interviewed. If a prescribing clinician was unwilling or ineligible, another prescriber from the list was randomly selected. In total, the researchers contacted 43 prescribers. One of these was not eligible because she no longer prescribed for PTSD patients. Of the remaining 42, 8 failed to respond to the e-mail invitation and 8 declined to participate due to lack of time, resulting in 26 participants and a 62% participation rate. Demographic and background information for the participants is presented in Table 1. All subjects gave their informed consent for inclusion before they participated in the study. The study was conducted in accordance with the Declaration of Helsinki, and the protocol was approved by the Dartmouth Committee for the Protection of Human Subjects and the White River Junction VA Research and Development Committee (CPHS# 22745).

**Table 1:** Prescribing Clinicians’ Characteristics.Gender, professional background, treatment setting, and years in practice of the 26 prescribing clinicians in our sample.

Gender *n (%)*	
Female	13 (50)
Professional Background *n (%)*	
Psychiatrist	13 (50)
Primary Care Staff Physician	6 (23)
Nurse Practitioner	2 (8)
Physician Assistant	3 (11)
Clinical Pharmacist	2 (8)
Primary treatment setting *n (%)*	
Primary Care	9 (35)
Mental Health	14 (53)
Integrated Care Setting	2 (8)
Residential Unit	1 (4)
Years Working for VHA	
*M (SD)*	9 (7.9)
Years in Current Position	
*M (SD)*	4.4 (3.9)

Half of the participants were psychiatrists, 23% primary care physicians, 11% physician’s assistants, and 8% each nurse practitioners and clinical pharmacists. After complete description of the study, we obtained participants’ written informed consent.

### 2.3. Procedures 

Two clinical psychologists, trained in qualitative interviewing, conducted the interviews over the telephone from August 2011 through February 2012. The psychologists alternated roles, with one serving as the lead interviewer and the other serving as back-up, taking notes and ensuring all topics were covered. A research assistant observed all interviews. The interviews were semi-structured; we followed the natural flow of the interview and asked follow-up questions when appropriate. Interviewers began by asking prescribing clinicians to describe their overall management plan when they first meet a Veteran with posttraumatic stress symptoms. Interview topics most pertinent to this study included strategies for making decisions about PTSD care, knowledge and perceptions of recommended psychotherapies for PTSD, access to EBPs, and barriers and facilitators to Veterans receiving EBPs in their facilities. The researchers audio recorded all interviews and professional transcriptionists produced verbatim transcripts. The research staff performed quality checks prior to analyses.

### 2.4. Data Analysis

The research team reviewed verbatim transcripts and identified broad themes to serve as top-level codes. Using inductive and deductive analysis with constant comparison [22] and methods Dr. Parker and colleagues developed for similar qualitative studies [23,24], the three members of the interview team each coded one-third of the transcripts and reviewed 100% of each other’s work. Then researchers resolved coding discrepancies through face-to-face meetings. The researchers created sub-codes by reviewing the quotes associated with each top-level code. Each interviewer took one-third of the quotes and applied the sub-codes. A secondary coder reviewed 25% of the sub-codes and discrepancies were discussed and resolved. Atlas.ti V6.2 qualitative data management software was used to assist with the coding and analysis.

## 3. Results

### 3.1. Overview

Several key themes emerged from our interviews, summarized in Table 2. The most commonly reported barrier to EBPs was lack of access. Another emerging theme was the role of prescribing clinicians in managing care for Veterans, for example, through discussing-or not discussing- psychosocial interventions and making-or not making-referrals for EBPs. Some prescribing clinicians had incorrect knowledge and erroneous beliefs about recommended psychotherapies that constituted a barrier to EBP adoption. Alternatively, a number of prescribing clinicians were able to describe facilitators and strategies to promote EBP adoption.

**Table 2:** Barriers and Facilitators to EBP Adoption from the Perspectives of Prescribing Clinicians. Prescribing clinicians’ response themes related to barriers and facilitators of EBPs for PTSD. The most commonly identified barrier was limited access to EBPs in their facilities. Prescribing clinicians also lacked knowledge in other areas and held some misperceptions that may delay or deter EBPs. Commonly identified facilitators included having enough providers trained in EBPs, having mental health providers integrated into medical clinics, seeing success after EBPs, and leadership support.

**Barriers**	
Believe access to EBPs is limited at their facilities	Insufficient number of MH clinicians generally and those trained in EBPs for PTSD on-site MH providers do not have time to be trained/supervised or to delivery weekly individual EBP
Lack of knowledge	Unaware of what EBPs their facilities offer Unaware of which therapies are evidenced based (e.g., individual trauma-focused cognitive behavioral therapies such as PE and CPT, EMDR, SIT) and which are not (e.g., non-specific group treatments) Misconceptions about which patients would benefit from EBPs and the need to “prepare” patients Misperceptions that EBPs are too expensive or time-consuming
Perceptions of patients’ attitudes:	Do not believe patients will engage in EBPs Believe patients prefer psychopharmacology treatments to EBPs
**Facilitators**	
Sufficient numbers of MH clinicians trained in EBPs on-site Seeing success in patients following EBPs MH clinicians embedded or integrated into PC clinics Strong leadership support and team resource sharing	

Note: EBP = Evidence-based psychotherapy; PE = Prolonged Exposure; CPT = Cognitive Processing Therapy; EMDR = Eye Movement Desensitization and Reprocessing; SIT = Stress Inoculation Training; MH = mental health; PC = primary care.

### 3.2. Perceived Access to EBPs: Barrier to Adoption

Limited access was the most commonly reported barrier to EBPs. Specifically, many prescribing clinicians reported that there were too few specialty MH therapists available, particularly those trained in PTSD EBPs. Sometimes EBPs were not available on-site. Further, many facilities did not provide sufficient protected time for therapists to receive training and supervision in the treatments. Nor did therapists’ schedules allow for the delivery of weekly, albeit time-limited sessions that the EBPs require:

“We try but we have a, kind of a shortage of psychologists that are available to do that (individual cognitive behavioral therapy). And so they can get in but not very frequent, not frequent enough to really be as effective as what is recommended. For me, I had first gotten trained in CPT (Cognitive Processing Therapy) 3 years ago was the first time, but it was just sheer patient caseload. Now as I told you, I’m supposed to be 50% clinical. And I have a caseload of over 130 people that I do everything for.” (Lead Nurse, Outpatient MH)

Lack of therapists trained in EBPs and limited time to deliver the individual therapies may be the reason group treatments are offered in so many facilities. A few prescribing clinicians referred Veterans to groups rather than individual treatment out of necessity, rather than lack of knowledge regarding the efficacy of these therapies. For example, according to one Behavioral Health Clinical Pharmacist, “I think that part of the reason that so many of our patients are shuttled into the groups initially is because we don’t have enough providers to give individual therapy”.

### 3.3. Prescribing Clinicians as Care Managers

Prescribing clinicians are often the first providers to encounter PTSD patients. Prescribing clinicians are often in charge of making referrals for psychosocial interventions and they are highly respected by patients and colleagues. For these reasons, prescribing clinicians play an important role in care decisions for Veterans. As one Physician’s Assistant in a general MH clinic described: “So I have all of these potential resources that I will eventually try to sort to match the individual”. When asked about their overall care plan, few prescribing clinicians described fully informing patients about all psychosocial services available and subsequently making collaborative decisions about care. Rather, they promoted pharmacological interventions first, usually an antidepressant, and sometimes described beliefs and practices that delayed or deterred what might be appropriate referrals to highly beneficial EBPs. For example, one prescribing clinician did not refer for psychosocial interventions immediately because of a belief that patients do not want immediate referrals:

“And so I lay the groundwork that they will be seeing me again if they want to, and then I probably would not be referring them on right then because I think people don’t like that very well.” (Nurse Practitioner, Outpatient MH).

Additionally, prescribing clinicians reported “stabilizing” patients with medication, which can take up to 2 months, before referring them to psychosocial interventions, although there is no evidence that such a practice improves outcomes:

“So once I get them somewhat stabilized with the use of medication, with beginning stress management, with giving them some early education, we’re going to start talking about evidence-based therapies if it seems like this is somebody who can do that.” (Lead Nurse, Outpatient MH)

Prescribing clinicians also believed that it was important to assess and build patient “readiness” to engage in EBPs before making referrals:

“So usually it’s like just make sure the assessment’s there, and trying to gauge kind of like readiness for treatment. Also, I try to do a little assessment about the Veteran’s ability just to kind of focus on memories related to their trauma, because that usually tells me how ready they might be, or how much work we’ve got to do to get them ready for evidence-based treatment.” (Staff Psychiatrist, MH)

Although this belief seems intuitively reasonable, there is little evidence that prescribing clinicians are able to devote sufficient time to substantially increase readiness or improve patients’ utilization of psychotherapies.

### 3.4. Knowledge and Perceptions of EBPs: Barriers to Adoption

As expected, PC prescribers were more likely than MH prescribers to not know what psychosocial interventions were available at their facilities and held more misperceptions about EBPs for PTSD. Surprisingly, however, even some MH prescribers were confused about the specific types of psychotherapies available and were uncertain what took place in treatment sessions. One MH staff psychiatrist observed: “I can’t tell you what they’re actually doing in their sessions, whether it’s PTSD or exposure or what, but I think they get better”.

In contrast to the PTSD guideline, which recommends individual therapies as the first line treatment for PTSD, many prescribing clinicians thought group therapy, usually described as supportive, educational, or skills-based, was best practice:

“I’ve certainly seen that on our PTSD Unit, of Veterans who come in, say they’re not taking any meds. I’m like, okay, no problem. You’re going to be getting group therapy here. This is the best treatment for PTSD.” (Medical Director for PTSD Unit, and Staff Psychiatrist)

Referrals to groups were common despite several prescribing clinicians stating that Veterans did not want to attend groups. Some prescribing clinicians referred Veterans to individual treatment only if the Veteran did not want group, with the ultimate goal of getting the Veteran into group treatment:

“Some of them clearly they don’t like the idea of group, they’re not going for group at the beginning. So sometimes what I’m doing is try to get them to start individual and then from there, whoever is doing the individual might encourage them to move into a group setting.” (Staff Psychiatrist, MH)

One possibly inaccurate assumption was that most patients do not want trauma-focused psychotherapy. “There are some access problems but the biggest access problem is that they (patients) don’t want to go”. (Staff Physician, PC). Some prescribing clinicians also doubted the feasibility of individual EBPs, which they deemed too expensive and time-consuming. Others also had misperceptions about the type of patient who would benefit from the psychotherapies (e.g., believed EBPs are only for the most severe patients):

“If they’re really, really, really bad, I mean like they’re in and out of ER all the time and in and out of locked ward all the time. Then if they’re, willing to go the distance, they’ll put them into Prolonged Exposure. But it’s really, really, really time-consuming.” (Director, OEF/OIF/OND Clinic)

### 3.5. Perceived Facilitators and Specific Strategies to Promote EBP Adoption

Despite the presence of significant barriers to EBPs, we found that where EBPs were readily available, many prescribing clinicians spoke positively about them. Some also spoke about the advantages of integrated MH specialists in their clinics, as well as the importance of leadership support and team resource sharing. Specific strategies believed to promote engagement in EBPs also emerged.

An important facilitator was the availability of well-trained psychotherapists and systems that allowed for EBP delivery:

“We’re fortunate in this clinic, we do have four psychologists and one social worker, so I do have people to refer to and they (patients) can get in quickly.” (Staff Psychiatrist)

“Most of them (therapists) are trained in CBT (cognitive behavioral therapy) and most of them use that anyway. So the social workers have the ability to schedule like a 90-min session every week with someone for example, to really do the PE (Prolonged Exposure) right.” (Staff Psychiatrist)

When their patients actually had the opportunity to undergo EBPs, many prescribing clinicians were impressed with effectiveness of these treatments, which we assume would lead to continued referrals for EBPs. As one staff psychiatrist (MH) stated “Oh yeah, sometimes (patients experience remission), especially with PE. I’m very impressed with PE”. Another example from a Lead Nurse in outpatient MH:

“The Vietnam Veteran Cognitive Processing Therapy (CPT) group, and I’m just astonished at these guys, they’ve been dealing with this forever, they’re pretty hopeless. And they change this fast, so I’m very impressed with that.”

The presence of MH specialists integrated into PC clinics was also particularly helpful:

“That’s the beauty of having that psychologist; part of what their purpose is being with us, quick access. He tries to refer them to that (PTSD clinic) but I’ll occasionally have somebody that either doesn’t want to go or somebody that had gone and had, you know, bad experiences so they don’t want to go any more. And then he’s (psychologist) been willing to work with those, you know, individually and it’s actually very nice to have.” (PC physician)

In addition, strong leadership support for EBPs, as well as resource sharing and discussion of EBPs during team meetings facilitated adoption. For example, one Chief of Psychiatry (MH) stated “Our Program Chief is a psychologist and he’s very good at making sure that they all follow the evidence base, so I think that’s going pretty well”. Sharing of resources among staff was also identified as useful:

“Some of the worksheets that we used in CPT (Cognitive Processing Therapy), the challenging questions, obviously the A-B-C (Activating Event, Belief, Consequence), everybody knows about that one, but challenging questions, that sort of thing, clinicians have talked about some of those pieces and in our larger team meetings.” (Lead Nurse, Outpatient MH)

Finally, prescribing clinicians reported specific strategies they believed promoted EBPs in their facilities, although it is unknown whether these practices actually increase utilization. Prescribing clinicians described building patient engagement and readiness for EBPs in two ways: First, by developing trust and providing education about PTSD and EBPs during their own appointments; and second, by referring patients to classes designed to engage patients in services before referring for EBPs. As one Lead Nurse in Outpatient MH put it:

“I would be telling them about our PTSD education group, which has really been a wonderful place for people to be able to kind of put their toe in the water without feeling too much pressure, we get really large numbers to that group, someone could come in and kind of sit in the back and not have to say anything if they don’t want to, and there’s going to be no pressure but they begin to get some education and begin to get a sense of not being so alone.”

## 4. Discussion

The current study adds a unique perspective on EBP adoption by examining the perspectives of prescribing clinicians, who, within VA, play a large role in helping Veterans with PTSD make decisions about their care. Lack of access to EBPs was the most commonly reported barrier. A seemingly sufficient number of therapists in VA have received initial trainings in the EBPs Prolonged Exposure and Cognitive Processing Therapy [7] to meet the needs of Veterans with PTSD. However, therapist turnover, role changes, lack of motivation, or lack of protected time for EBP consultation and delivery (*i.e.*, weekly individual sessions) could all contribute to under-use of EBPs. The VA must make major organizational changes to give therapists time to not only attend the EBP trainings and ongoing consultation. Perhaps more importantly, VA must give therapists time to deliver these weekly individual treatments, which are brief (*i.e.*, 8–16 weeks) and therefore allow many patients to receive the treatments quickly. The VA could also consider providing incentives to therapists who provide EBPs (e.g., compensation time, bonus). Further, VA could invest research funds into developing and evaluating EBPs delivered in group format. Referring Veterans to groups rather than individual treatment was described as a necessity in some facilities. If certain group treatments are shown to be as effective as individual EBPs, this could help solve access problems.

When EBPs are available, it is essential that prescribing clinicians hold accurate beliefs about these treatments and their availability within their facilities, patient preferences, and the sustained treatment gains and cost-effectiveness. Clinical prescribers also need to know how to effectively assess patient readiness to engage in EBPs and how to facilitate effective referrals.

To increase clinical prescribers’ knowledge of the VA/DoD Clinical Practice Guideline for PTSD (e.g., individual EBPs received Level A rating; group treatments received Level C rating), VA could distill these guidelines into brief, accessible, user-friendly documents and disseminate them more effectively. This information would need to be updated regularly. For example, new research findings released after the PTSD guideline and after our interviews were conducted demonstrate that EBPs for PTSD have sustained and larger effect sizes than medications [9,10]. Information on EBPs could also be provided on a local level: Embedded or integrated mental health providers could inform prescribers about EBP availability within their facilities. A number of prescribing clinicians, particularly PC providers, reported that the integration of MH providers into PC clinics increased access. Many VA and civilian clinics have adopted integrated care models that increase MH utilization for those with PTSD [25]. Embedded behavioral health providers who are well-trained in effective treatment options can serve as the optimal facilitators not only to best PTSD care, but for MH conditions generally [26]. An integrated care model could promote collaboration and communication between therapists and prescribing clinicians in both directions. Therapists could inform prescribing clinicians about EBPs and availability in their clinic, streamline the referral process, and support Veterans with psychotropic medication decisions and compliance. Prescribing clinicians could also then support the work of EBPs and inform therapists about best practice pharmacological interventions for PTSD.

Regarding patient preferences, prescribing clinicians may benefit from training in shared decision making models of care, where care decisions are made with, not for, patients. Some research suggests that Veterans with PTSD report more concerns about psychotropic medications than psychosocial interventions [27]. Moreover, people with trauma exposure and PTSD rate descriptions of EBPs as more desirable than first-line pharmacological interventions, and, when provided with their preference, benefit the most from psychosocial interventions [28]. Finally, research also suggests that time-limited EBPs for PTSD may be cost effective by reducing further MH utilization [29,30]. This information could be disseminated to clinical prescribers through formal VA dissemination pathways, or imparted by mental health providers within each respective facility.

Clinical prescribers could also be provided formal readiness for EBP assessment tools [31] rather than gauging readiness using their intuition. The brief time prescribing clinicians have for individual appointments, both within and outside VA, may not be sufficient to assess readiness or to prepare patients for EBPs. In addition, there is only limited evidence that engagement groups or classes increase utilization of EBPs. To increase EBP utilization, engagement groups could provide specific information about EBPs and follow up systematically with graduates using “warm” handoffs to EBP therapists. These, and other specific strategies used to increase utilization of EBPs, should be studied. Some clinics have developed local EBP referral tracking systems that systematically monitor whether EBPs for PTSD (and other conditions) have been offered to each Veteran. These should be broadly expanded in VA. To our knowledge, this is the first study to inquire about clinical prescribers’ perspectives on EBPs, providing insights into their referral decisions. Future research examining referral behaviors of clinicians with primarily pharmacological training to psychosocial evidence-based interventions is warranted.

This study has several limitations. First, the primary focus of the interviews was on factors that influenced pharmacological approaches to PTSD treatment but provided valuable information about factors contributing to dissemination of EBPs that we felt were critical to share. Second, we did not interview psychotherapists, and as such, do not know whether they hold the same perceptions, or misperceptions, about EBPs, and whether the perceived lack of access to EBPs in their facilities was accurate. We also did not interview case managers, patients, or family members. Future work on PTSD treatment recommendations would benefit from interviews with these stakeholders. Third, we are unable to formally compare the composition of professional backgrounds in our sample to that of all VA prescribing clinicians, across settings, providing care to Veterans with PTSD specifically. However, we believe it is reasonable to assume that our random sample represents those of the prescribing clinicians in their respective setting (PC* vs.* MH). Fourth, we are unable to discern whether non-responders (38%) differed from responders (62%) in any meaningful ways that could change the findings.

## 5. Conclusions

Our findings have important implications for PTSD care and beyond despite these limitations. Administrators and stakeholders, both within VA and the community, need to utilize mechanisms we and other researchers have identified to improve the implementation process so that every person has access to an EBP. Perhaps most importantly, patients and families need and deserve to be informed about effective treatments for PTSD and then afforded the opportunity to advocate for themselves.
